# Liver metastases in thyroid cancer: epidemiology, risk stratification and survival outcomes in the immunotherapy era

**DOI:** 10.3389/fimmu.2025.1624181

**Published:** 2025-07-25

**Authors:** Ming-Lu Xu, Yang-Yang Wang, Li-Ping Xie, Ning Ding, Jia-Jun Hui

**Affiliations:** ^1^ Department of Medical Oncology, Wuxi Huishan District People’s Hospital, Affiliated Huishan Hospital of Xinglin College, Nantong University, Wuxi, China; ^2^ Department of Otolaryngology, Wuxi Huishan District People’s Hospital, Affiliated Huishan Hospital of Xinglin College, Nantong University, Wuxi, China; ^3^ Department of Medical Oncology, Suzhou Dushu Lake Hospital, The Fourth Affiliated Hospital of Soochow University, Suzhou, China

**Keywords:** thyroid cancer, liver metastases, SEER database, nomogram, risk prediction, survival analysis, immunotherapy

## Abstract

**Purpose:**

Liver metastases in thyroid cancer are rare but fatal, with poorly defined risk profiles and survival outcomes. This study aimed to characterize epidemiology, risk factors and outcomes of this disease using a population-based approach, further explore the potential impact of the immunotherapy era on the prognosis of these patients.

**Methods:**

Data on 116,801 thyroid cancer cases from SEER program (2010-2021) were analyzed. The clinicopathological features of patients with and without liver metastases were compared. Logistic regression analyses were employed to identify the predictors for liver metastases, while survival determinants were determined using Cox regression models. The predictive nomogram was developed for liver metastasis risk assessment, validated using concordance index, calibration curves, receiver operating characteristic (ROC) curves, and decision curve analysis (DCA). In addition, we further compared the prognostic outcomes of these patients in the immunotherapy era.

**Results:**

The prevalence of liver metastasis in thyroid cancer was 0.22% (95%CI 0.20%-0.25%), predominantly in medullary thyroid carcinoma (MTC) and anaplastic thyroid carcinoma (ATC). MTC exhibited the highest risk of metastasis (OR=35.7, 95%CI 24.1–52.8). The nomogram for liver metastasis risk (C-index=0.98) demonstrated robust discriminatory ability and clinical utility. The median overall survival (OS) was 6.0 months (95%CI 4.0–8.0), with survival rates of 38.1% at 1 year, 28.3% at 3 years, and 16.5% at 5 years. Patients with ATC and rare histology types experienced significantly shorter survival. No statistically significant difference in mOS and median cancer-specific survival (mCSS) of these patients between the pre- and post-immunotherapy eras were observed (P>0.05 for both).

**Conclusion:**

This study establishes the first population-based predictive framework for liver metastases in thyroid cancer, underscoring risk stratification and survival. These findings also highlight the critical need to optimize survival outcomes for this aggressive metastatic phenotype in immunotherapy era.

## Introduction

Thyroid cancer (TC) has emerged as the fastest-growing malignancy of the endocrine system, with a widespread and persistent increase in TC incidence (1). According to the incidence and mortality estimates from GLOBOCAN 2022, it is projected that approximately 1,100,000 new TC cases and 91,000 TC-related deaths will occur in 2050, respectively (2). The incidence of thyroid cancer varies substantially by geographic location, particularly among the female population and in higher-income countries. Among the major pathological types of TC, approximately 90% are papillary thyroid carcinoma (PTC), 4% are follicular thyroid carcinoma (FTC), 2% are medullary thyroid carcinoma (MTC), and 1% are anaplastic thyroid carcinoma (ATC) (3). While the differentiated subtypes (PTC and FTC) typically exhibit indolent behavior, the prognosis significantly worsens when distant metastases occur, particularly in patients with ATC and MTC patients (4, 5). Although the incidence of distant metastases at the initial diagnosis of TC is only 4%, the prevalence of distant metastases may increase to 33% in high-risk patients during the course of treatment and follow-up (6). The presence of distant metastases is the most significant prognostic factor associated with poor outcomes (7, 8).

Current epidemiological investigations have identified lung and bone as the predominant metastatic sites for TC (9, 10). Liver metastases from TC are rare, with a reported frequency of approximately 0.5%, and population-based incidence estimates vary across single-center reports (6, 11, 12). Liver metastases tend to occur during the terminal phase of TC and represent a serious clinical event. Shah DH et al. reported that survival after diagnosis of liver metastases among 11 patients with well-differentiated thyroid carcinoma ranges from 1 to 60 months (11). Brient C et al. found that the median survival after the diagnosis of liver metastases in a series of 14 patients with differentiated thyroid cancer was 17.4 months (6). However, the accurate prognosis for these patients has not been clearly defined due to the rarity of this condition. Therefore, the clinicopathological characteristics and treatment of thyroid cancer patients with liver metastases warrant urgent investigation, as the current understanding of this disease is primarily based on small sample studies or case reports. The Surveillance, Epidemiology, and End Results (SEER) program is one of the most comprehensive and authoritative population-based cancer registries globally (13, 14). Its longitudinal design, initiated in 1973, and standardized reporting protocols ensure robust collection of rare oncologic events with minimal selection bias. In the present study, we aimed to explore the incidence, risk factors, and prognosis of TC patient with liver metastases by utilizing the data from these patients recruited in the SEER database between 2010 and 2021.

Immunotherapy has emerged as a cornerstone in the management of advanced solid tumors (15). However, patients with liver metastases often exhibit suboptimal responses to immune checkpoint inhibitors, likely due to the liver’s unique immunosuppressive microenvironment (16). Notably, a landmark study conducted in 2019 was the first to evaluate the PD-1 inhibitor (pembrolizumab) in refractory thyroid cancer, demonstrating both anti-tumor efficacy and manageable toxicity (17). Nevertheless, the clinical utility of immunotherapy in thyroid cancer patients with liver metastases remains entirely unexplored. To address this critical knowledge gap, we conducted an indirect temporal comparison of survival outcomes by stratifying patients based on diagnosis timelines relative to the immunotherapy era (pre-2019 vs. post-2019), allowing us to preliminarily assess the real-world impacts of evolving therapeutic paradigms.

## Materials and methods

### Data sources

The SEER database, maintained by the National Cancer Institute (NCI), encompasses approximately 48% of the U.S. population across geographically diverse regions. SEER prospectively collects data on cancer incidence, clinicopathological characteristics, treatment modalities, and survival outcomes. The database (Incidence - SEER Research Plus Data, 17 registries, Nov 2023 Sub, 2000–2021) was queried to identify thyroid cancer patients with liver metastases diagnosed between 2010 and 2021. This retrospective analysis of de-identified, publicly available data from the SEER database qualifies for exemption from institutional ethics review.

### Patients enrollment

The patients were identified based on the tumor locations (Site recode ICD-O-3/World Health Organization [WHO] 2008: Thyroid) and the status of liver metastases, as explicitly defined by the “Extent of Disease. SEER Combined Mets at DX-liver (2010+)”. To ensure analytical precision, the inclusion was restricted as follows: 1) thyroid cancer as the only one primary tumor; 2) known status of liver metastases (Yes or No); 3) sufficient information on survival time and follow-up for conducting survival analysis.

### Variables collection

For each patient, the following variables were identified: 1) Demographic characteristics, including age, sex, race, and marital status; 2) Year of diagnosis, based on 2019 clinical trial that firstly reported the exploratory use of PD-1 inhibitor in advanced thyroid cancer (17), we operationally defined patients diagnosed with thyroid cancer liver metastases after 2019 as the immunotherapy era cohort. 3) Clinicopathological features, including pathological type, AJCC-T/N/M staging at initial diagnosis, and the presence or absence of lung, bone, or brain metastases; 4) Therapeutic details, encompassing surgery, radiotherapy, and chemotherapy; and 5) Survival data, including survival time and survival status. The primary focus of the epidemiological investigation was the incidence of liver metastases, while the endpoints of the survival analysis included overall survival (OS) and cancer-specific survival (CSS). OS was defined as the interval from the initial diagnosis to death from any cause or the last follow-up, while CSS was defined as the interval from the initial diagnosis to death specifically caused by thyroid cancer or the last follow-up.

### Statistical analysis

Demographic, clinicopathological, and treatment variables were defined as categorical variables. Age was stratified into clinically meaningful categories: less than 55 years, 55 to 65 years, and greater than 65 years. Histological subtypes were classified as PTC, FTC, MTC, ATC, or other to account for rare variants. Chi-squared (χ²) tests were used to analyze the differences in categorical variables between thyroid cancer patients with and without liver metastases. To identify risk factors for liver metastasis, a two-step analytical approach was employed. Initially, univariable logistic regression analyses were performed, followed by the incorporation of significant risk factors into a multivariate logistic regression analysis to identify independent risk factors, along with their odds ratio (ORs) and 95% confidence interval (CI). Subsequently, the independent risk factors were included in the development of a risk predictive nomogram. The established nomogram was evaluated using Harrell’s C-index and the time-varying area under the curve (AUC) to confirm their predictive performance and discrimination accuracy (18, 19). To assess the predictive accuracy of the nomogram, calibration curves were generated to evaluate the agreement between predicted probabilities and observed outcomes (20). Additionally, decision curve analysis (DCA) was conducted to quantify the clinical applicability of the nomogram model by calculating the net benefit across a range of threshold probabilities (21).

For survival analysis, the Kaplan-Meier curve was utilized to assess the OS and CSS of thyroid cancer patients with liver metastases. The log-rank test was employed to evaluate differences in OS and CSS stratified by various factors. The univariate Cox analysis was used to identify the prognostic factors associated with OS and CSS. Nubmer needed to treat (NNT) was used as a clinically statistic to compare the post-immuno era effectiveness among thyroid cancer patients with liver metastases. NNT represented the number of patients needed to treat with a new method, instead of the standard method, for one patient to benefit. NNT for survival benefit (OS and CSS) for at least 12 months after diagnosis were calculated. Statistical analyses were performed using R software (version 4.3.1; R Foundation for Statistical Computing, Vienna, Austria). A two-sided significance threshold of P < 0.05 was applied to all tests, with results considered statistically significant if they fell below this threshold.

## Results

### Prevalence of liver metastases in thyroid cancer patients

A total of 116,801 thyroid cancer patients were identified in the SEER database from 2010 to 2020. Among these patients, 261 cases presented with liver metastases at initial diagnosis. The prevalence of liver metastases in thyroid cancer is 0.22% (95%CI 0.20%-0.25%).

### Clinicopathological characteristics of thyroid cancer patients with liver metastases

Patients with liver metastases exhibited distinct clinicopathological profiles compared with those without liver metastases. As shown in **Table 1**, patients with liver metastases were significantly older, with 54.1% (141/261) aged ≥65 years, compared to 16.3% (19,034/116,540) in the non-liver metastases group (p<0.01). A male predominance was also observed, with 49.4% (129/261) versus 23.6% (27,463/116,540) in the non-liver metastases group (p<0.001). Histologically, non-PTC pathological types dominated these cases, particularly MTC (31.8% [83/261]) and ATC (19.5% [51/261]), which were 21-fold and 24-fold more frequent, respectively, than in those without liver metastases, respectively (both p<0.001). These patients presented with aggressive tumor phenotypes characterized by advanced AJCC-T stage (T3-4: 62.8% vs 22.4%) and a higher likelihood of lymph nodal involvement (N1: 63.6% vs 25.7%, p<0.001). Notably, 58.2% of liver metastasis cases exhibited concurrent lung metastases, 49.0% had bone metastases, and 6.9% demonstrated brain metastases, all of which were significantly higher than non-metastatic patients (all p<0.001). Regarding treatment regimens, patients with liver metastases were less likely to receive surgery (38.7% vs 96.2%) and radioisotope therapy (7.7% vs 37.6%), while they were more likely to receive chemotherapy (38.3% vs 1.0%) and external radiotherapy (29.5% vs 2.4%) (all p<0.001).

**Table 1:** The demographic and clinicopathological characteristics of thyroid cancer patients with and without liver metastases.

Characteristics	TC patients (N=116,801)	TC patients without Liver metastases (N=116,540)	TC patients with Liver metastases (N=261)	Prevalence of Liver metastases (% with 95%CI)
Age				
<55y	75,440 (64.6%)	75,378 (64.7%)	62 (23.7%)	0.08, 0.06-0.10
55-65y	22,186 (19.0%)	22,128 (19.0%)	58 (22.2%)	0.26, 0.20-0.34
>65y	19,175 (16.4%)	19,034 (16.3%)	141 (54.1%)	0.74, 0.63-0.87
Gender				
Female	89,209 (76.4%)	89,077 (76.4%)	132 (50.6%)	0.15, 0.13-0.18
Male	27,592 (23.6%)	27,463 (23.6%)	129 (49.4%)	0.47, 0.4.-0.56
Race				
White	91,699 (78.5%)	91,506 (78.5%)	193 (73.9%)	0.21, 0.18-0.24
Black	7,763 (6.6%)	7,734 (6.7%)	29 (11.1%)	0.37, 0.26-0.53
Other	15,110 (12.9%)	15,072 (12.9%)	38 (14.6%)	0.25, 0.18-0.34
Unknown	2,229 (19.1%)	2,228 (1.9%)	1 (0.4%)	0.04, 0.01-0.25
Marital status				
Married	68,067 (58.3%)	67,929 (58.3%)	138 (52.9%)	0.20, 0.17-0.24
Single	28,122 (24.1%)	28,068 (24.1%)	54 (20.7%)	0.19, 0.15-0.25
Unknown	6,935 (5.9%)	6,924 (5.9%)	11 (4.2%)	0.16, 0.09-0.29
Other	13,677 (11.7%)	13,619 (11.7%)	58 (22.2%)	0.42, 0.32-0.54
Pathological type				
PTC	104,730 (89.7%)	104, 673 (89.8%)	57 (21.8%)	0.05, 0.04-0.07
FTC	7,668 (6.6%)	7,645 (6.6%)	23 (8.8%)	0.30, 0.20-0.45
MTC	1,787 (1.5%)	1,704 (1.5%)	83 (31.8%)	4.64, 3.76-5.72
ATC	1,027 (0.9%)	976 (0.8%)	51 (19.5%)	4.97, 3.80-6.48
Other	1,589 (1.4%)	1,542 (1.3%)	47 (18.0%)	2.96, 2.23-3.91
AJCC-T				
T1	65,785 (56.3%)	65,768 (56.4%)	17 (6.5%)	0.03, 0.02-0.05
T2	20,951 (17.9%)	20,932 (18.0%)	19 (7.3%)	0.09, 0.06-0.14
T3	21,724 (18.6%)	21,673 (18.6%)	51 (19.5%)	0.23, 0.17-0.30
T4	4,519 (3.9%)	4,406 (3.8%)	113 (43.3%)	2.50, 2.08-3.00
TX	3,822 (3.3%)	3,761 (3.2%)	61 (23.4%)	1.60, 1.25-2.05
AJCC-N				
N0	80,358 (68.8%)	80,301 (68.9%)	57 (21.8%)	0.07, 0.05-0.09
N1	30,090 (25.8%)	29,924 (25.7%)	166 (63.6%)	0.55, 0.47-0.64
NX	6,353 (5.4%)	6,315 (5.4%)	38 (14.6%)	0.60, 0.44-0.82
AJCC-M				
M0	113,448 (97.1%)	113,448 (97.3%)	0 (0.0%)	0.00
M1	2,660 (2.3%)	2,399 (2.1%)	261 (100%)	9.81, 8.74-11.0
MX	693 (0.6%)	693 (0.6%)	0 (0.0%)	0.00
Lung metastases				
Yes	1,688 (1.4%)	1,536 (1.3%)	152 (58.2%)	9.00, 7.73-10.5
No	115,047 (98.5%)	114, 945 (98.6%)	102 (39.1%)	0.09, 0.07-0.11
Unknown	66 (0.1%)	59 (0.1%)	7 (2.7%)	10.6, 5.24-20.3
Bone metastases				
Yes	905 (0.7%)	777 (0.6%)	128 (49.0%)	14.1, 12.0-16.6
No	115,861 (99.2%)	115,736 (99.3%)	125 (47.9%)	0.11, 0.09-0.13
Unknown	35 (0.0%)	27 (0.0%)	8 (3.1%)	22.9, 12.1-39.0
Brain metastases				
Yes	151 (0.1%)	133 (0.1%)	18 (6.9%)	11.9, 7.67-18.1
No	116,622 (99.8%)	116,390 (99.8%)	232 (88.9%)	0.20, 0.18-0.23
Unknown	28 (0.0%)	17 (0.0%)	11 (4.2%)	39.3, 23.6-57.6
Surgery				
Yes	112,168 (96.0%)	112,067 (96.2%)	101 (38.7%)	0.09, 0.07-0.11
No	4,633 (4.0%)	4,473 (3.8%)	160 (61.3%)	3.45, 2.96-4.02
Radiation				
Radioisotopes	43,806 (37.5%)	43,786 (37.6%)	20 (7.7%)	0.05, 0.03-0.08
Radiotherapy	2,887 (2.5%)	2,810 (2.4%)	77 (29.5%)	2.67, 2.14-3.32
None/Unknown	70,108 (60.0%)	69,944 (60.0%)	164 (62.8%)	0.23, 0.20-0.27
Chemotherapy				
Yes	1,215 (1.0%)	1,115 (1.0%)	100 (38.3%)	8.23, 6.81-9.91
No	115,586 (99.0%)	115,425 (99.0%)	161 (61.7%)	0.14, 0.12-0.16

### Independent risk factor for liver metastases in thyroid cancer patients

**Figure 1:**
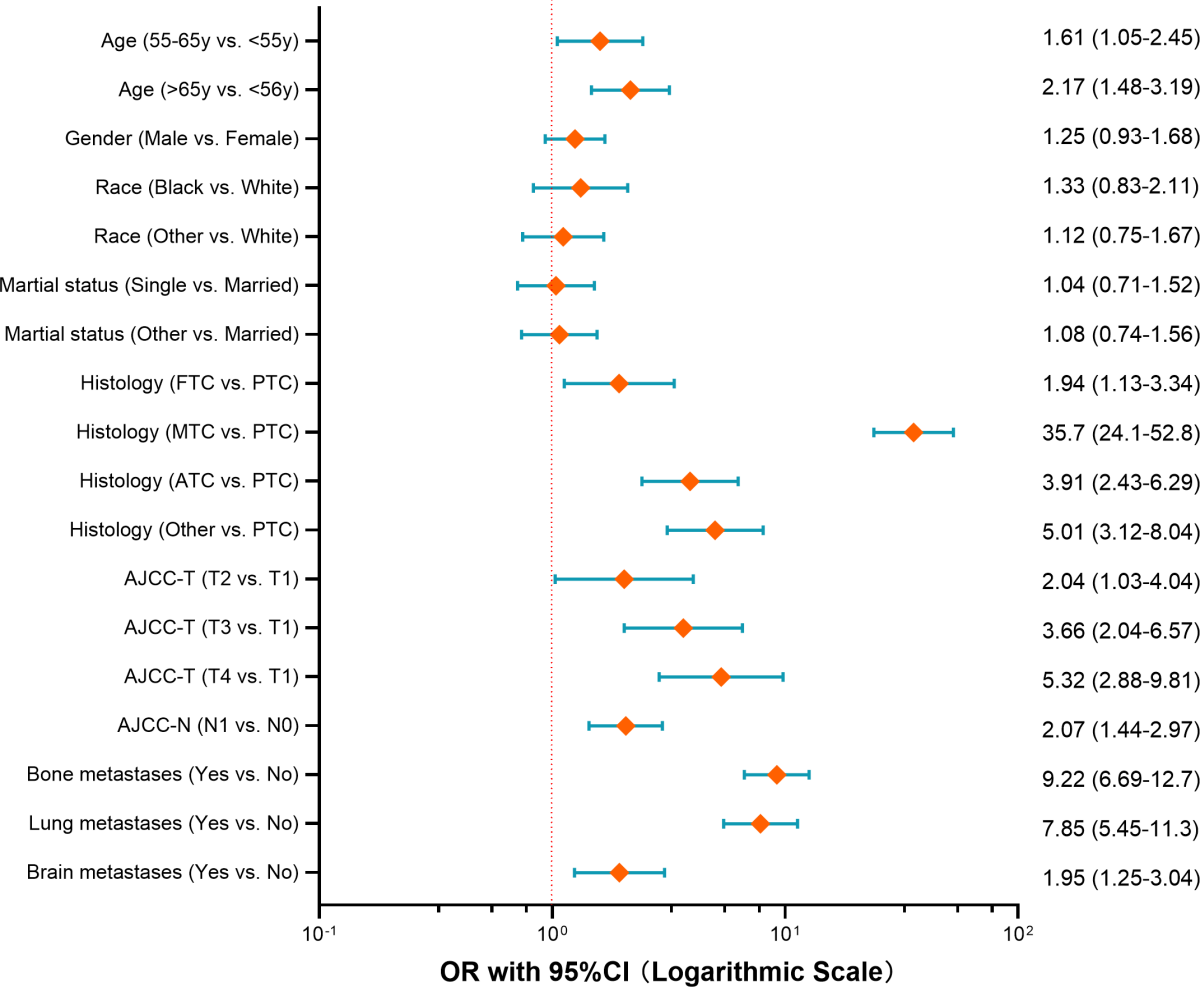
Forest plot of risk factors for developing liver metastases among thyroid cancer (OR with 95%CI axis in logarithmic scale).

To identify the independent risk factors for liver metastases in patients with thyroid cancer, multivariable logistic regression analysis was conducted. The results indicated that age, histology, AJCC-T stage, lymph node metastases, and metastases to the lung, bone, or brain were the independent risk factors (**Figure 1**). Among these, MTC histology emerged as the strongest predictor for liver metastasis (OR=35.7, 95% CI 24.1-52.8), followed by distant metastases to other organs: lung (OR=7.85), bone (OR=9.22), brain (OR=1.95).

### Liver metastases predictive nomogram

**Figure 2:**
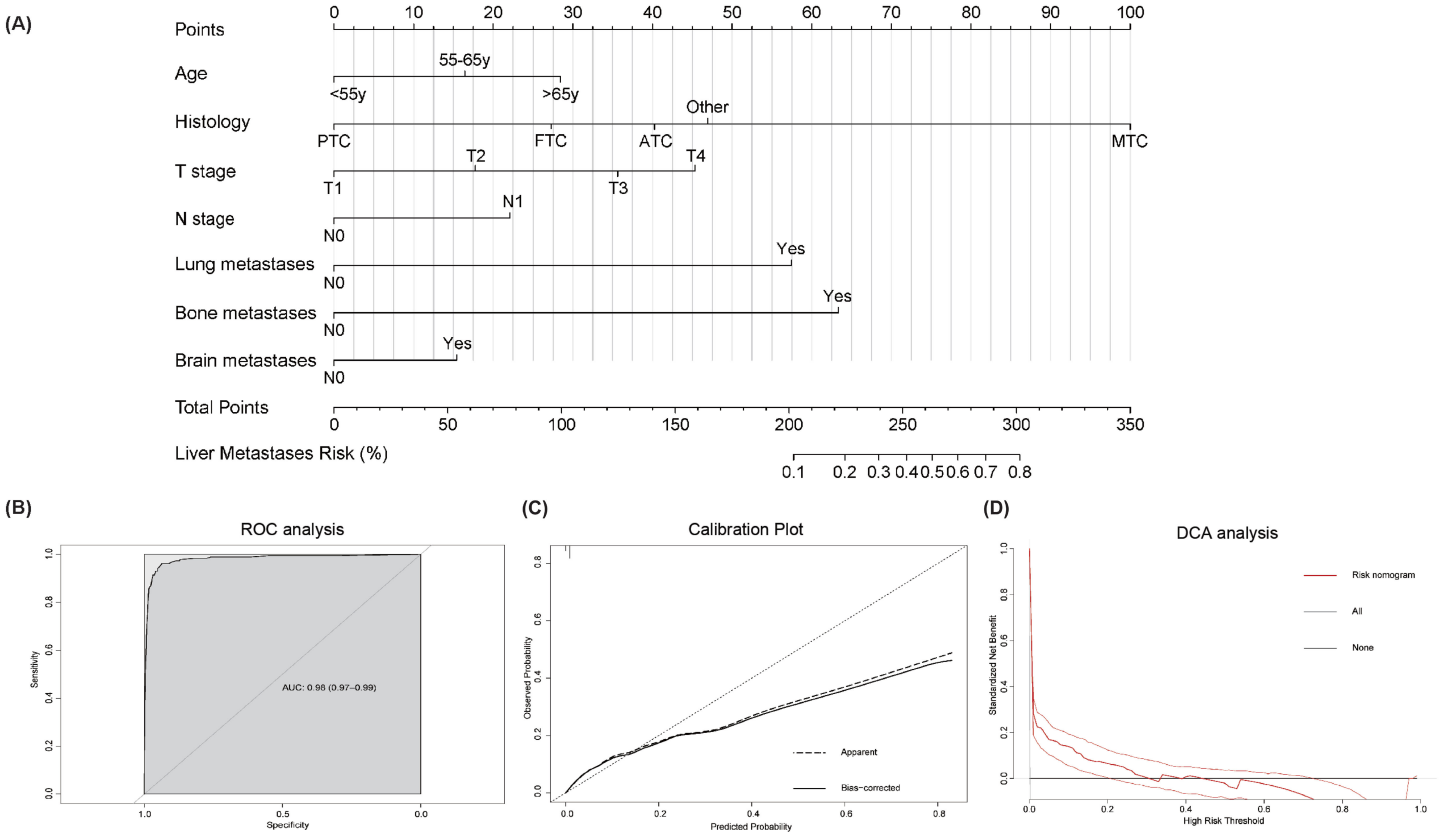
Risk-predictive Nomogram to assess the risk of liver metastases in thyroid cancer patients **(A)**. Receiver operating characteristic (ROC) analysis **(B)**, calibration plot **(C)**, and decision curve analysis (DCA) **(D)** are used to assess the performance of the established nomogram. To use this nomogram: Locate individual patient value on each variable axis, draw vertical lines to the points scale, sum these points, and align the total with the outcome axis to obtain the predicted probability.

A risk prediction nomogram model for thyroid cancer patients at risk of developing liver metastases was constructed using the independent predictive factors identified from the above multivariate logistic regression analysis. As shown in **Figure 2**, the C-index for this risk-predictive nomogram was 0.98, with histopathological subtype being the most significant contributor to the model. The ROC analysis revealed that the area under the curve (AUC) value for this risk-predictive nomogram is 0.98 (95% CI 0.97–0.99), demonstrating excellent discriminatory capacity (**Figure 2B**). Calibration curves indicated optimal agreement between predicted and observed probabilities (**Figure 2B**). Decision curve analysis confirmed the clinical utility value of this model in clinical practice (**Figure 2B**).

Illustrative examples of risk-predictive nomogram application:

Example 1: A 70-year-old patient with medullary thyroid carcinoma (MTC), classified as T4N1 stage with lung metastasis. Applying the corresponding point values from the nomogram: age (>65 years): 28.5 points, histology (MTC): 100 points, T-stage (T4): 45.5 points, N-stage (N1): 22 points, lung metastasis (Yes): 57.5 points, bone metastasis (No): 0 points, brain metastasis (No): 0 points. The total score was calculated as 253 points. According to the nomogram’s probability scale, this corresponds to an estimated 40% probability of developing liver metastasis.
Example 2: A 50-year-old patient with medullary thyroid carcinoma (MTC), classified as T3N1 stage with lung and bone metastases. Applying the corresponding point values: age (<55 years): 0 points, histology (MTC): 100 points, T-stage (T3): 35.5 points, N-stage (N1): 22 points, lung metastasis (Yes): 57.5 points, bone metastasis (Yes): 63.5 points, brain metastasis (No): 0 points. The total score was 278.5 points. According to the nomogram’s probability scale, this corresponds to an estimated 63% probability of developing liver metastasis.

### Survival analysis

**Figure 3:**
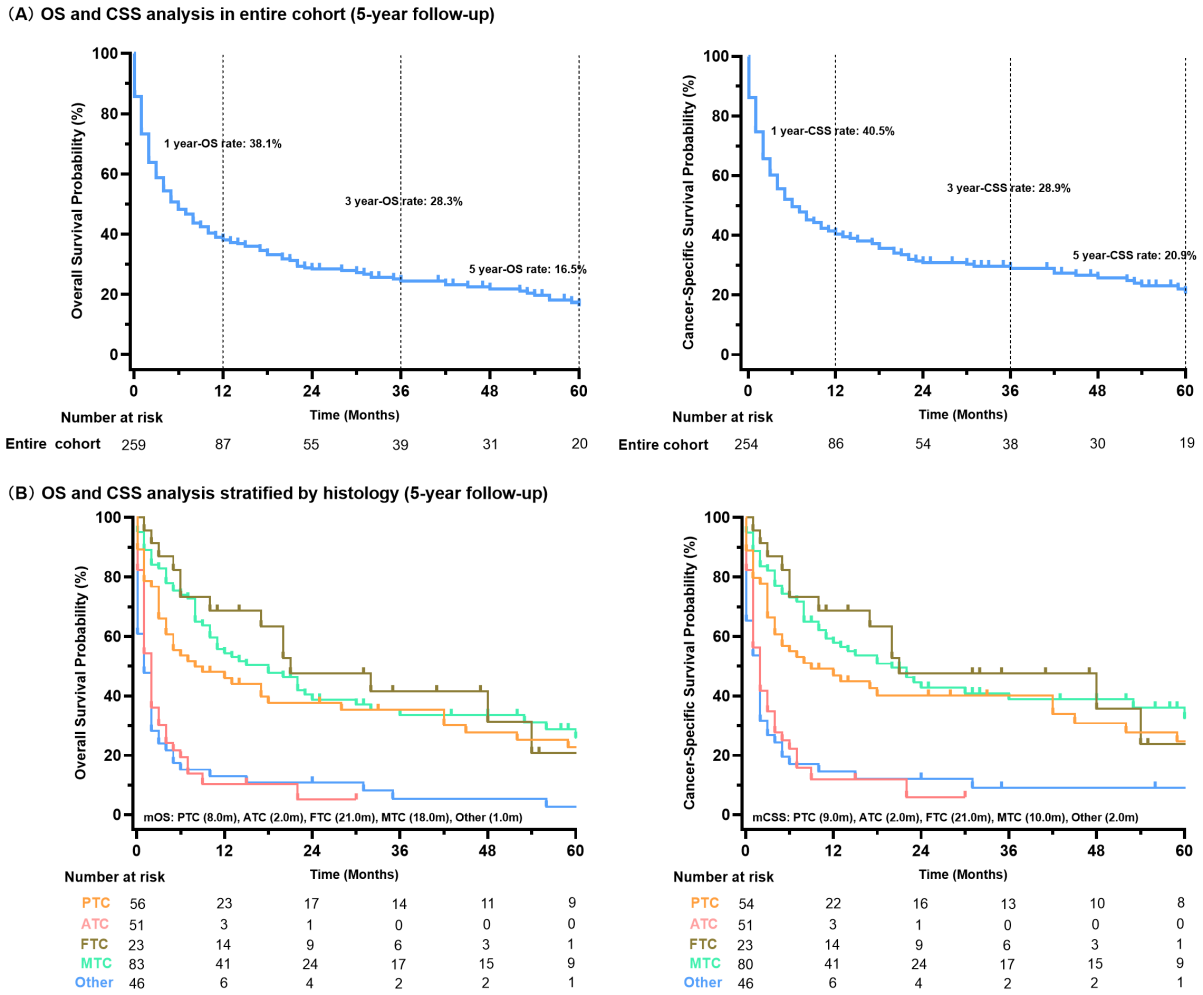
Survival analysis of all thyroid cancer patients with liver metastases **(A)**; stratified by histology type **(B)**.

The survival outcomes for thyroid cancer with liver metastases were significantly poor (**Figure 3A**). The cohort exhibited a median overall survival (mOS) of 6.0 months (95%CI 4.0 - 8.0), with survival rates of 38.1% at 1 year, 28.3% at 3 years, and 16.5% at 5 years. The CSS analysis revealed similar trends, with a median CSS (mCSS) of 6.0 months (95%CI 4.0-10.0) and corresponding 1-, 3-, and 5-year CSS rates of 40.5%, 28.9%, and 20.9%, respectively. Survival analysis stratified by pathological type indicated that patients with ATC and other rare types of thyroid cancer with liver metastases had significantly shorter mOS and mCSS than MTC, PTC and FTC (P<0.01 for all, **Figure 3B**).

### Prognostic factors for thyroid cancer patients with liver metastases

The univariable Cox regression analysis identified 10 candidate variables potentially associated with OS and CSS. The results demonstrated strong associations between histopathological aggressiveness and deterioration in survival, with ATC emerging as the most significant predictor of poor OS (HR=2.72, 95%CI 1.75-4.22). Notably, the presence of lung metastases was associated with a 111% increased risk of mortality (HR=2.11, 95%CI 1.55-2.85). Conversely, anti-tumor therapy interventions exhibited protective effects: surgical resection reduced the risk of death by 68% (HR=0.32, 95%CI 0.23-0.44), radioisotope therapy by 73% (HR=0.27, 95%CI 0.14-0.52), and chemotherapy by 29% (HR=0.71, 95%CI 0.53-0.94) (**Table 2**). Similar observations were noted in the CSS analysis, where ATC histology continued to exert a significant adverse impact in the presence of lung metastases, while anti-tumor therapy maintained its protective associations (**Table 3**).

**Table 2:** Univariate Cox regression analysis for OS among thyroid cancer patients with liver metastases.

Characteristics	Univariate Cox analysis	
	HR with 95%CI	P value
Age (vs. <55y)		
55-65y	1.26 (0.81-1.96)	0.30
>65y	1.92 (1.33-2.76)	<0.01
Gender (vs. Female)		
Male	0.89 (0.67-1.17)	0.41
Race (vs. White)		
Black	0.95 (0.61-1.47)	0.80
Other	0.82 (0.54-1.25)	0.36
Unknown	1.70 (0.24-12.2)	0.60
Marital status (vs. Married)		
Single	1.02 (0.71-1.48)	0.90
Other	1.64 (1.17-2.30)	<0.01
Unknown	0.92 (0.46-1.83)	0.81
Pathological type (vs. PTC)		
FTC	0.71 (0.40-1.29)	0.26
MTC	0.78 (0.52-1.16)	0.22
ATC	2.72 (1.75-4.22)	<0.01
Other	2.69 (1.75-4.12)	<0.01
AJCC-T (vs. T1)		
T2	1.15 (0.51-2.60)	0.74
T3	0.99 (0.50-1.96)	0.97
T4	2.02 (1.08-3.79)	0.03
TX	1.98 (1.03-3.79)	0.04
AJCC-N (vs. N0)		
N1	0.91 (0.65-1.28)	0.61
NX	1.46 (0.94-2.26)	0.09
Lung metastases (vs. No)		
Yes	2.11 (1.55-2.85)	<0.01
Unknown	1.96 (0.85-4.56)	0.11
Bone metastases (vs. No)		
Yes	1.36 (1.01-1.81)	0.04
Unknown	2.04 (0.94-4.44)	0.07
Brain metastases (vs. No)		
Yes	1.82 (1.06-3.10)	0.03
Unknown	2.48 (1.30-4.74)	<0.01
Surgery (vs. No)		
Yes	0.32 (0.23-0.44)	<0.01
Radiation (vs. No/unknown)		
Radioisotopes	0.27 (0.14-0.52)	<0.01
Radiotherapy	0.95 (0.70-1.29)	0.76
Chemotherapy (vs. No)		
Yes	0.71 (0.53-0.94)	0.02

**Table 3:** Univariate Cox regression analysis for CSS among thyroid cancer patients with liver metastases.

Characteristics	Univariate Cox analysis	
	HR with 95%CI	P value
Age (vs. <55y)		
55-65	1.27 (0.79-2.04)	0.32
>65	1.94 (1.32-2.87)	<0.01
Gender (vs. Female)		
Male	0.88 (0.66-1.16)	0.37
Race (vs. White)		
Black	0.85 (0.53-1.37)	0.51
Other	0.81 (0.52-1.26)	0.35
Unknown	1.73 (0.24-12.44)	0.58
Marital status (vs. Married)		
Single	1.05 (0.71-1.55)	0.80
Other	1.72 (1.20-2.45)	0.00
Unknown	0.93 (0.45-1.92)	0.84
Pathological type (vs. PTC)		
FTC	0.71 (0.38-1.31)	0.27
MTC	0.73 (0.48-1.13)	0.16
ATC	2.54 (1.61-4.03)	<0.01
Other	2.50 (1.58-3.93)	<0.01
AJCC-T (vs. T1)		
T2	1.22 (0.52-2.88)	0.65
T3	1.00 (0.49-2.05)	1.00
T4	2.07 (1.07-3.99)	0.03
TX	1.82 (0.91-3.63)	0.09
AJCC-N (vs. N0)		
N1	0.88 (0.62-1.26)	0.48
NX	1.26 (0.78-2.02)	0.34
Lung metastases (vs. No)		
Yes	2.19 (1.59-3.03)	<0.01
Unknown	2.17 (0.93-5.05)	0.07
Bone metastases (vs. No)		
Yes	1.46 (1.07-1.99)	0.02
Unknown	1.90 (0.82-4.39)	0.13
Brain metastases (vs. No)		
Yes	1.93 (1.11-3.36)	0.02
Unknown	2.62 (1.37-5.00)	<0.01
Surgery (vs. No)		
Yes	0.33 (0.24-0.46)	<0.01
Radiation (vs. No/unknown)		
Radioisotopes	0.29 (0.14-0.57)	<0.01
Radiotherapy	0.99 (0.72-1.36)	0.94
Chemotherapy (vs. No)		
Yes	0.71 (0.52-0.97)	0.03

### Prognosis of thyroid cancer patients with liver metastases in the era of immunotherapy

**Figure 4:**
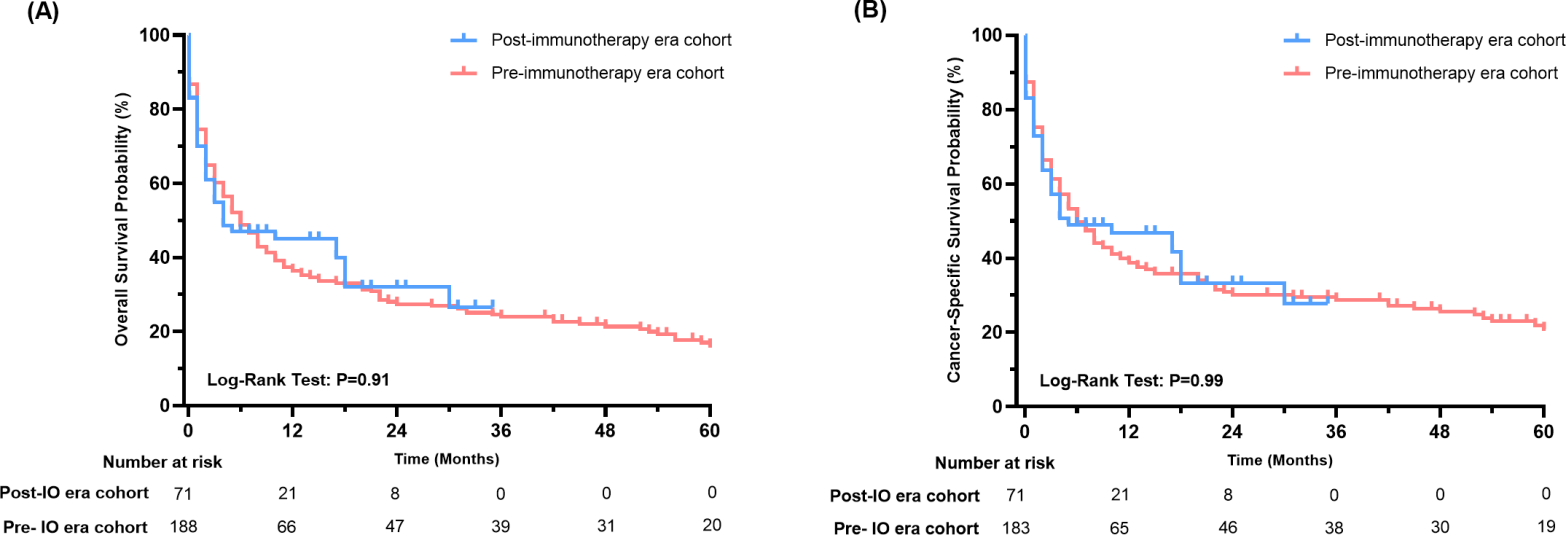
Survival analysis of thyroid cancer patients with liver metastases in the immunotherapy era and pre-immunotherapy era (**A**: OS, **B**: CSS).

Further, we compared the OS and CSS of thyroid cancer patients with liver metastases before and after 2019. Out of 261 patients, 71 were classified as being in the immunotherapy era. Although survival analysis revealed no statistically significant differences in OS or CSS between the pre- and post-immunotherapy era cohorts (P>0.05 for both, **Figure 4**), the Kaplan-Meier curves indicated a distinct trend favoring the immunotherapy era subgroup, particularly beyond 12 months (12-months OS rate: 45.0% vs. 36.3%; 12-months CSS rate: 46.9% vs 38.7%). NNT for 12-months OS benefit from post-immunotherapy era is 11 patients, while NNT for 12-months CSS is 12 patients. This suggests potential clinical benefits in a subset of patients.

## Discussion

This study firstly provides critical insights into the epidemiology, risk factors, clinicopathological features, and clinical outcomes of thyroid cancer patients with liver metastases, leveraging the strengths of a large population-based cohort. The observed prevalence of 0.22% for liver metastases at initial diagnosis underscores the importance of population-based studies in comprehensively understanding this rare metastatic event. Furthermore, this study significantly enhances the knowledge regarding liver metastases among thyroid cancer patients by quantifying risk factors and prognostic indicators, as well as establishing the models for predicting the development of liver metastases. Meanwhile, we conducted the first comprehensive analysis of survival outcomes in thyroid cancer patients with liver metastases during the immunotherapy era, suggesting the potential existence of immunotherapy-responsive subpopulations that warrants further exploration.

Our findings both confirm and challenge existing literature on liver metastases in thyroid cancer. Worldwide, approximately 64.63% of TC cases occur in populations under 55 years old, while nearly 82.99% of TC-related deaths occur in populations aged 55 years and older (2). Elderly patients often have a poor prognosis or aggressive tumor histology (22, 23). In consistent with this, our study also demonstrated that elderly thyroid cancer patients are more likely to develop liver metastases. The biological behavior of MTC is less favorable than that of differentiated thyroid cancer (PTC, FTC), about 10% of patients presenting with distant metastases at initial diagnosis (24, 25). The association between MTC and liver metastasis corroborates previous studies that reported liver involvement in 45% of advanced MTC cases (26). In line with these reported data, our population-level analysis provides stronger evidence for the unique metastatic pattern of MTC, with 4.64% of MTC patients developing liver metastases. Although ATC patients had highest prevalence of liver metastases (4.97%), MTC emerged as the most significant risk factor for developing liver metastases after adjusting for confounding factors. Furthermore, it is understandable that patients with a greater tumor burden or advanced staging often have a higher risk for liver metastases, including advanced T stage/N stage and the presence of lung, bone, or brain metastases.

Our analysis revealed a distinct prognostic paradigm in thyroid cancer patients with liver metastases. Contrary to the behavior typically observed in solid tumors (27–29), nodal involvement (N-stage) was not identified as a prognostic factor in Cox analysis, regardless of OS or CSS. Instead, therapeutic interventions, including surgery, chemotherapy, and radiotherapy, emerged as the primary determinants of survival, consistent with prior investigations (5, 30). This paradigm shift likely reflects the exceptionally aggressive biology of this disease, where advanced stage or tumor burden at diagnosis renders localized tumor characteristics prognostically irrelevant, while treatment response becomes the critical factor associated with survival. The results also confirmed a strong association between histologic subtype and survival outcomes in thyroid cancer patients with liver metastases, aligning with the aggressive biology of ATC. Notably, ATC patients with liver metastases exhibited a significantly worse prognosis compared to those with differentiated subtypes (PTC/FTC). Intriguingly, patients with rare histologic subtypes exhibited survival curve that paralleled those of ATC (1-year OS: 10.3% vs. 13.0%). This suggests that these entities share a comparable level of biological aggressiveness when metastasizing to the liver (31, 32). This finding underscores the urgent need for early therapeutic intervention in these rare subtypes. In contrast, MTC, which is associated with a risk of liver metastases, demonstrated survival outcomes similar to those of PTC and FTC (1-year OS: 54.4% for MTC vs. 68.6% for FTC vs. 46.0% for PTC), despite its high propensity for liver metastasis. This paradox may reflect MTC’s relatively indolent growth kinetics compared to ATC. Collectively, these observations highlight that survival in thyroid cancer with liver metastases is influenced not only by the occurrence of metastasis, but predominantly by the intrinsic malignant potential of the primary histology. Additionally, distinct treatment strategies across thyroid cancer histologic subtypes may contribute to the observed survival differences. For instance, the different prevalence of target gene alterations (e.g., BRAF in PTC, RET in MTC) could directly impact the availability and utilization of corresponding targeted therapies, which would be also a potential underlying factor in these survival differences.

Notably, the steep decline in survival curves highlighted a critical therapeutic window within the first 12 months post-diagnosis, during which over 60% of mortality events occurred. Both OS and CSS curves exhibited a characteristic biphasic pattern—an initial rapid decrease phase followed by prolonged stabilization among a small subset of survivors. The potential heterogeneity in disease aggressiveness across different histologies may contribute to this observation, while treatment approaches represent another significant factor among patients. Consistent with finding related to lung metastases in thyroid cancer (30), surgery, and chemotherapy emerged as important prognostic factors associated with improved survival. Our results suggest that radioiodine therapy is the primary determinant of survival in thyroid cancer patients with liver metastatic. Most of the patients with liver involvement also present with metastases to lymph nodes, bones, and lungs, necessitating systemic therapies. The observed survival benefit from chemotherapy (HR=0.71) in our cohort may reflect advancements in systemic treatment options. In addition to radiation and chemotherapy, surgery-though often underutilized in metastatic cancer patients-could provide the clinical benefits for thyroid cancer patients with liver metastases. Consistent with previous data, the resection of liver metastases in thyroid cancer remains a viable option, even in cases involving large lesions (33). One consideration for this strategy should be based on an analysis of the feasibility of performing a radical and safe resection. In addition to surgery, locoregional radiological treatments may also be the preferred option when feasible (26, 34). The survival advantage associated with multimodal therapy underscores the clinical urgency of early intervention.

Immunotherapy has emerged as a pivotal therapeutic strategy for advanced thyroid cancer (15, 35). However, heterogeneous PD-L1 expression levels across histological subtypes correlate with varying treatment responses. Prior studies have identified tumor-infiltrating immune subsets enriched in PD-1^+^CD4^+^ T cells, and PD-1^+^CD8^+^ T cells within a subset of thyroid tumors, suggesting potential sensitivity to PD-1/PD-L1 blockade (36). Initial evidence from a phase Ib trial (NCT02628067) demonstrated that pembrolizumab has a manageable safety profile and exhibits antitumor activity in certain patients with advanced differentiated thyroid cancer (17). A recent phase II trial further validated the clinical activity of dual immune checkpoint inhibition (nivolumab + ipilimumab) in aggressive thyroid cancers (37). Despite these advances, the efficacy of immunotherapy in thyroid cancer patients with liver metastases remains unexplored. Challenges in conducting prospective trials for this rare metastatic model prompted our innovative approach: stratifying patients based on diagnosis timelines relative to the immunotherapy era (pre-2019 vs. post-2019) to indirectly assess therapeutic impacts. Kaplan-Meier analysis revealed no significant difference in survival. Nevertheless, landmark analysis at 12 months demonstrated a nearly 10% absolute OS and CSS improvement in the immunotherapy-era cohort, with potential survival benefit observed. These findings suggest a clinically relevant subpopulation may derive durable benefit from ICIs, although validation through prospective biomarker-guided trials is necessary.

Another significant contribution of this study is the development and validation of predictive tools that assess the risk of liver metastasis. The metastasis prediction nomogram incorporates six clinically accessible variables, allowing clinicians to estimate individual probabilities of liver metastasis during the initial evaluation. The high-performance nomogram (C-index=0.982) also fill a critical gap in risk stratification tools for thyroid cancer. Current guidelines do not provide specific recommendations for monitoring hepatic metastasis; however, our models identify high-risk subgroups (e.g., MTC patients with lung metastases) who may benefit from intensified imaging examinations.

Like other population studies based on the SEER database, several limitations warrant consideration (22, 38). First, the SEER database lacks detailed information including performance scores, molecular data (e.g., RET mutations in MTC, BRAF status in PTC) and treatment details (e.g., dosage and scheduling), which may confound the observed associations between histology and outcomes. Second, due to the rarity of liver metastases in thyroid cancer patients, external validation using a real-world cohort could not be conducted. Third, information on distant metastasis has only been documented since 2010 in the SEER database, which may limit the sample size. Another limitation lies in the inability of the SEER database to describe treatment modalities during the immunotherapy era, including the use of immunotherapy, which may bring potential biases in interpreting the findings. The survival differences between these two cohorts (pre- and post- immunotherapy era) should be cautiously explained. However, this study represents the first indirect exploration on the impact of immunotherapy on thyroid cancer liver metastases, offering a novel perspective in this understudied area.

In conclusion, this study establishes liver metastases as a rare yet devastating complication of thyroid cancer, characterized by distinct risk patterns and prognostic implications across histological subtypes. The developed prediction models provide clinically actionable tools for risk assessment, while the observed therapeutic benefits highlight the potential value of early intervention in these patients. These findings also highlight the critical need to optimize survival outcomes for this aggressive metastatic phenotype in immunotherapy era.

## Data Availability

The original contributions presented in the study are included in the article/supplementary material. Further inquiries can be directed to the corresponding authors.
